# Impact of polyethylene microplastics exposure on kallikrein-3 levels, steroidal-thyroidal hormones, and antioxidant status in murine model: protective potentials of naringin

**DOI:** 10.1038/s41598-024-74637-5

**Published:** 2024-10-10

**Authors:** Samuel Abiodun Kehinde, Tolulope Peter Fatokun, Abosede Temitope Olajide, Sarva Mangala Praveena, Adewale Allen Sokan-Adeaga, Adegbola Philip Adekunle, Dalia Fouad, Marios Papadakis

**Affiliations:** 1 Biochemical/Environmental Toxicology Laboratory, Faculty of Basic Medical Sciences, Ajayi Crowther University, Oyo, Nigeria; 2 Department of Drug Toxicology and Safety Pharmacology, Faculty of Life Sciences, University of Bradford, Bradford, UK; 3 Cell and Signaling Laboratory, Department of Biomedical Science, Faculty of Medicine and Health Sciences, Universiti Putra Malaysia, UPM Serdang, Serdang, Selangor Darul Ehsan 43400 Malaysia; 4 Department of Environmental and Occupational Health, Faculty of Medicine and Health Sciences, Universiti Putra Malaysia, UPM Serdang, 43400 Serdang, Selangor Darul Ehsan Malaysia; 5 Department of Environmental Health Science, Faculty of Basic Medical Science, Ajayi Crowther University, Oyo, Nigeria; 6 Department of Environmental Health Science, Oyo State College of Health Sciences and Technology, Ibadan, Nigeria; 7 Department of Zoology, College of Science, King Saud University, PO Box 2455, Riyadh, 11451 Saudi Arabia; 8 Department of Surgery II, University Hospital Witten-Herdecke, University of Witten-Herdecke, Heusnerstrasse 40, 42283 Wuppertal, Germany

**Keywords:** Microplastics, Polyethylene microplastics, Naringin, Oxidative stress, Endocrine disruption, Hormones, Biochemistry, Cell biology, Chemical biology, Biomarkers, Endocrinology

## Abstract

The widespread presence of microplastics in the environment has raised significant concerns regarding their potential impact on human and animal health. Among various microplastic types, polyethylene microplastics (PE-MPs) are particularly prevalent due to the extensive use in packaging and consumer products. Exploring the uncharted therapeutic potentials of naringin, this study delves into its mitigating effects on disruptions in kallikrein-3 levels, steroidal-thyroidal hormone balance, and antioxidant defense triggered by PE-MPs exposure, paving the way for novel interventions in environmental toxin-induced endocrine and oxidative stress disorders. Male Wistar rats (*n* = 24) were randomly grouped into four: Control, PE-MPs (1.5 mg/kg), PE-MPs + NAR (1.5 mg/kg PE-MPs + 100 mg/kg NAR), and NAR (100 mg/kg). Hormonal and antioxidant parameters were assessed after 28 days of exposure. PE-MPs exposure caused a significant increase(*p* < 0.005) in the level of kallikrein-3 (KLK-3) while it significantly reduces the levels of testosterone (TST), luteinizing hormone, thyroid stimulating hormone (TSH) and Free-triiodothyronine (fT3) and Total cholesterol (TChol) concentration. PE-MPs exposure also disrupted significantly (*p* < 0.005) antioxidant profile by down-regulating the activities of glutathione-S-transferase, catalase (CAT), superoxide dismutase (SOD) and reducing levels of glutathione (GSH) and ascorbic acid (AA) while concentration of malondialdehyde (MDA) levels were increased relative to control. However, the mitigating potentials of naringin on disruptions in hormonal and antioxidant profiles caused by PE-MPs exposure were demonstrated, as NAR normalized KLK-3, steroid, and thyroid hormone levels, cholesterol concentration, and enhanced antioxidant defense. This suggests that NAR is a promising protective agent against endocrine and oxidative damage induced by environmental contaminants such as microplastics.

## Introduction

The proliferation of plastic pollution has emerged as a significant environmental concern, with microplastics becoming a pervasive contaminant in various ecosystems. Microplastics, defined as plastic particles less than 5 mm in diameter, have been identified in a wide array of environmental media and matrices, including soil, water, and air, raising alarms about their potential impact on wildlife and human health^1–3^. Among these, polyethylene microplastics (PE-MPs) are particularly prevalent due to the extensive use of polyethylene in various consumer products and packaging materials and their persistence and ubiquity have raised profound concerns with respect to their impact on environmental and biological systems^4^. Microplastics can enter the environment through multiple pathways, including the degradation of larger plastic debris, wastewater effluents, and industrial processes. Once in the environment, they can be ingested by a wide variety of organisms, leading to potential physical and chemical impacts^5^. Polyethylene microplastics (PE-MPs) typically have a negative zeta potential, which influences their stability in suspension and interaction with other particles and organisms. The exact value varies with environmental factors like pH and salinity. In terms of shape, PE-MPs can be spherical (e.g., from personal care products) or irregular and fragmented due to environmental degradation. Shape affects their behavior in ecosystems, with irregular particles often having higher contaminant adsorption and different bioavailability compared to smooth, spherical MPs. Both zeta potential and shape are critical in determining the fate and impact of PE-MPs in the environment^6–9^.

Recent research has begun to unravel the complex interactions between microplastics and biological systems, particularly focusing on their ability to act as vectors for chemical contaminants and their potential to disrupt physiological processes. Endocrine disruption is one of the most concerning effects associated with microplastic exposure because the endocrine system, which includes glands and hormones regulating mood, metabolism, development, growth, and tissue function, is highly sensitive to exogenous chemicals^10^. Steroid hormones, such as testosterone, and thyroid hormones are crucial for maintaining homeostasis and normal physiological functions. Disruption in the levels of these hormones can lead to a cascade of adverse health effects, including reproductive issues, metabolic disorders, and developmental abnormalities.

Furthermore, exposure to microplastics raises significant concerns about oxidative stress. When the body’s antioxidant defense system and the production of reactive oxygen species (ROS) are out of balance, oxidative stress results. High ROS levels can cause inflammation, cellular damage, and a number of diseases^11^. Microplastics have been shown to induce oxidative stress, thereby exacerbating their harmful effects on biological systems^12^.

Phytochemicals are naturally occurring compounds in plants that offer various health benefits. Major classes include flavonoids, carotenoids, glucosinolates, and phenolic acids. Phytochemicals have antioxidant, anti-inflammatory, anticancer, and cardioprotective properties^13^. Naringin, a naturally occurring flavonoid, has been shown in various studies to possess anti-inflammatory, anti-apoptotic, antioxidant, anti-carcinogenic, anti-osteoporotic, and anti-ulcer properties. Additionally, naringin is noted for its role in lowering triglycerides and cholesterol levels, enhancing immune function, and improving antioxidant status. It has been shown to mitigate oxidative stress and modulate endocrine functions in various experimental models^14^. Several other studies has highlighted various therapeutic potentials of naringin^15–17^ .Therefore, naringin holds potential as a protective agent against the adverse effects of microplastic exposure.

There is paucity of information on the effects of PE-MPs on the endocrine system and the amelioration of its toxicity using phytotherapy. We aim to examine the impact of PE-MPs on the endocrine system and antioxidant status of Wistar rats. Specifically, we will examine the levels of key steroid and thyroid hormones and assess oxidative stress markers to determine if exposure to these microplastics disrupts hormonal balance and induces oxidative stress. Furthermore, we will evaluate the potential protective and ameliorative effects of naringin in mitigating these disruptions and oxidative stress associated with microplastic exposure.

## Materials and methods

### Chemicals

PE-MP (34–50 μm particle size, Cat No. 434272, CAS-No. 9002-88-4, Melting point 144 °C, Density 0.94 g/mL at 25 °C, Ultra-high molecular weight) and Naringin (CAS No. 10236-47-2, Molecular weight 580.53) were obtained from Sigma-Aldrich, Germany. The total cholesterol (TCHOL) kit was sourced from Cypress Diagnostics, Langdorp, Belgium. AK Scientific Inc., CA. supplied the reduced glutathione. The enzyme immunoassay (EIA) test kits for prostate-specific antigen (KLK-3), testosterone, LH, TSH and fT3 were purchased from BioCheck Inc., CA, USA. All other chemicals and reagents utilized were of analytical grade.

### Animals

The 180 ± 20 g male Wistar rats (8–12 weeks old) were kept in the university’s animal facility with conventional parameters, such as 22–25 °C temperature, 45 ± 5% humidity, and a 12-hour light/dark cycle. Food and water were freely available to the animals. They were kept on a regular rat pellet diet manufactured by Vita Feeds Nigeria Limited. To avoid cross-contamination, drinking water was only supplied in bottles with steel stoppers, and each experimental group was given a separate set of bottles.

### Experimental design

**Fig. 1:**
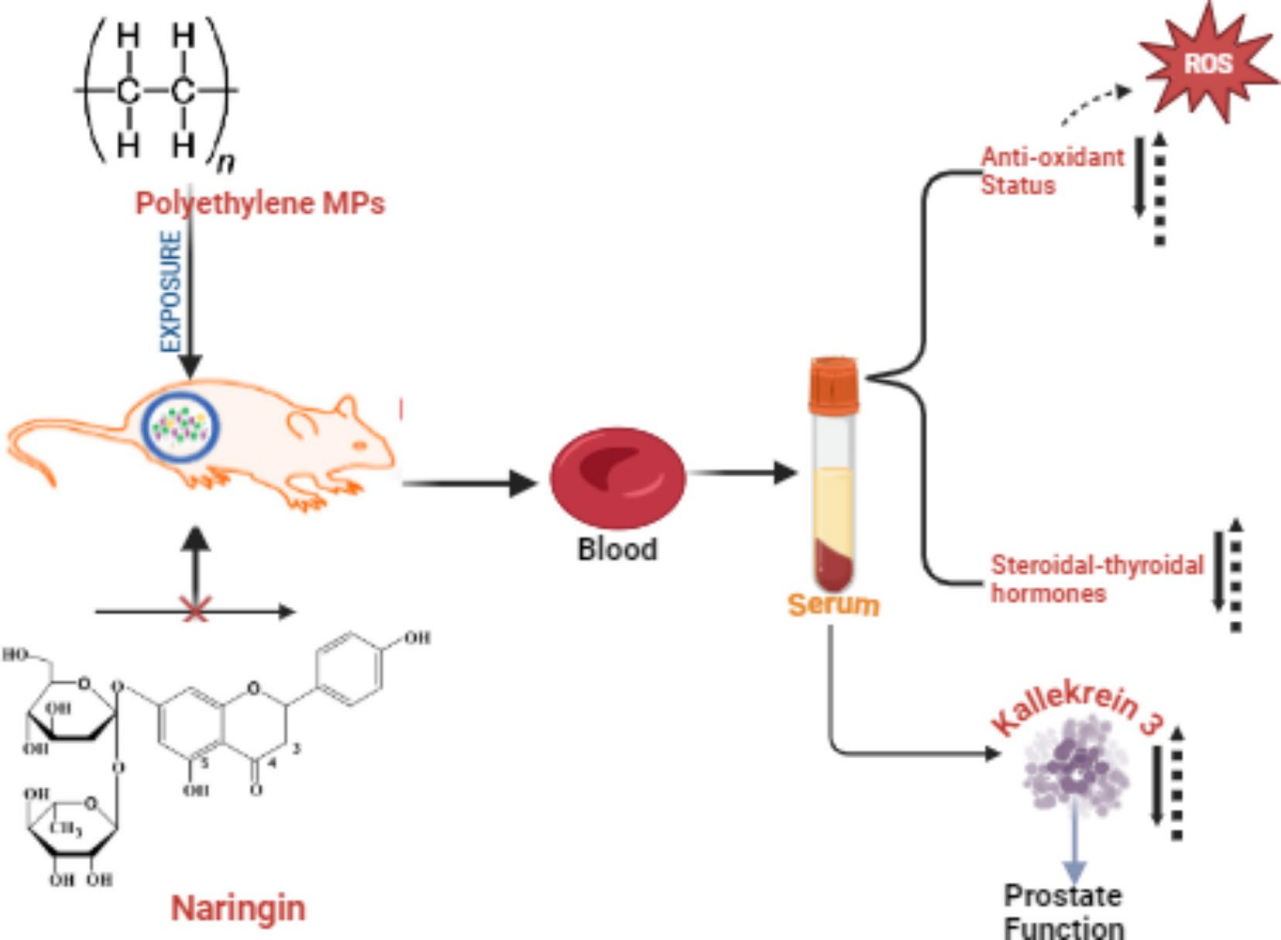
Graphical abstract (Created in Biorender.com).

Twenty-four rats were randomly divided into four groups (*n* = 6/group). The groups were exposed to the following treatments: the control group received 0.1 mL of normal saline; the PE-MP treated group received 1.5 mg/kg body weight of PE-MP orally; the PE-MP + Naringin treated group received 1.5 mg/kg body weight of PE-MP and 100 mg/kg body weight of Naringin; and the Naringin treated group received 100 mg/kg body weight of Naringin orally. Both PE-MP and Naringin were administered via oral gavage for 28 days (Fig. 1). The doses were selected based on previous studies^18–20^. All groups received PE-MPs using a 5 mL gavage needle directed to the pharyngeal region to minimize particle loss, ensuring effective uptake. Under reference number FNS/ERC/23/029EN, the Faculty Ethical Review Committee of the University accepted the experimental protocol confirming that all experiments followed relevant guidelines and regulations. The care was administered in adherence with accepted standards for the handling and care of laboratory animals and was carried out following ARRIVE guidelines.

### Sample collections and preparations

Twenty-four hours following the last dose, the rats were euthanized via Isoflurane (2–3%) induced anaesthesia in which the animal is placed in a sealed chamber connected to an isoflurane vaporizer which delivers 2–3% concentration of isoflurane mixed with air. The methods complied with international standards for the use and care of laboratory animals^21^ and carried out following ARRIVE guidelines. After being extracted from the abdominal artery, the blood samples were put in sterile, simple vials and allowed to stand for 20–30 min. After that, the samples were centrifuged (model *FC5816R*) at 4 °C for ten minutes at 3000 rpm. The collected serum was separated and aliquoted into sterile 1 mL Eppendorf tubes, which were kept at -18 °C until needed for further analysis (Fig. 1).

### Estimations of serum levels of TST, LH, fT3 , TSH, and KLK-3

Serum levels of KLK-3, LH, TSH, TST, and fT3 were measured using ELISA kits procured from BioCheck Inc., CA, USA. These assays rely on antibody-antigen reactions, performed by following the manufacturer’s instructions. The colour intensity developed in the assays—which directly correlates with concentrations in the test samples—was detected spectrophotometrically (model BC-660Plus) at 450 nm. By serially diluting the samples to match the dilutions of the standards included with the kits, the linearity of each ELISA assay was confirmed. With an average correlation coefficient (R^2^) of 0.89 ± 0.06, which is comparable with the standard curves, the samples showed satisfactory linearity.

### Determination of total cholesterol (TCHOL) concentration

The technique described in Cypress Diagnostics Kits, Langdorp, Belgium, was used to test serum total cholesterol (TCHOL). By hydrolysing the free cholesterol from the cholesterol esters with cholesterol esterase, cholesterol oxidase oxidizes the free cholesterol that has been liberated. Red quinonimine dye is created when 4-aminophenazone, phenol, and the resultant hydrogen peroxide (H_2_O_2_) combine. The amount of cholesterol present is directly correlated with the dye’s colour intensity.

### Determination of oxidative stress and antioxidant defense status

Using the technique outlined by Buege and Aust^22^, the concentration of malondialdehyde (MDA), an indicator of lipid peroxidation, was measured. The protocol described by Moron et al.^23^ was followed in order to quantify the serum levels of reduced glutathione (GSH). The activity of serum glutathione S-transferase (GST) was evaluated using the enzyme-catalysed reaction between glutathione and 1-chloro-2,4-dinitrobenzene, using the methodology outlined by Habig et al.^24^. The capacity of serum superoxide dismutase (SOD) to prevent adrenaline from self-oxidizing to adrenochrome at an alkaline pH formed the basis for the Misra and Fridovich^25^ technique of measuring SOD activity. The technique created by Sinha^26^ was used to measure the activity of serum catalase (CAT). Using the Folin-Ciocalteu method, as reported by Jagota and Dani^27^, ascorbic acid (AA) levels in the serum were determined. Using this approach, AA combines with the reagent to give a blue hue with maximal absorbance at 760 nm. Using bovine serum albumin (BSA) as the reference for the calibration curve, the total protein content of the samples was determined using the Bradford method^28^. Readings of absorbance were obtained at 595 nm.

### Statistical analysis

The reported values were expressed as mean ± standard deviation (SD). GraphPad Prism 5.0 was used to do One-Way Analysis of Variance (ANOVA) with Tukey’s post-hoc test. Statistical significance was determined by taking p-values less than 0.05.

## Results

### Effects of PE-MP and naringin on serum levels/concentration of KLK-3, TChol, TST, LH, TSH and fT3

**Fig. 2:**
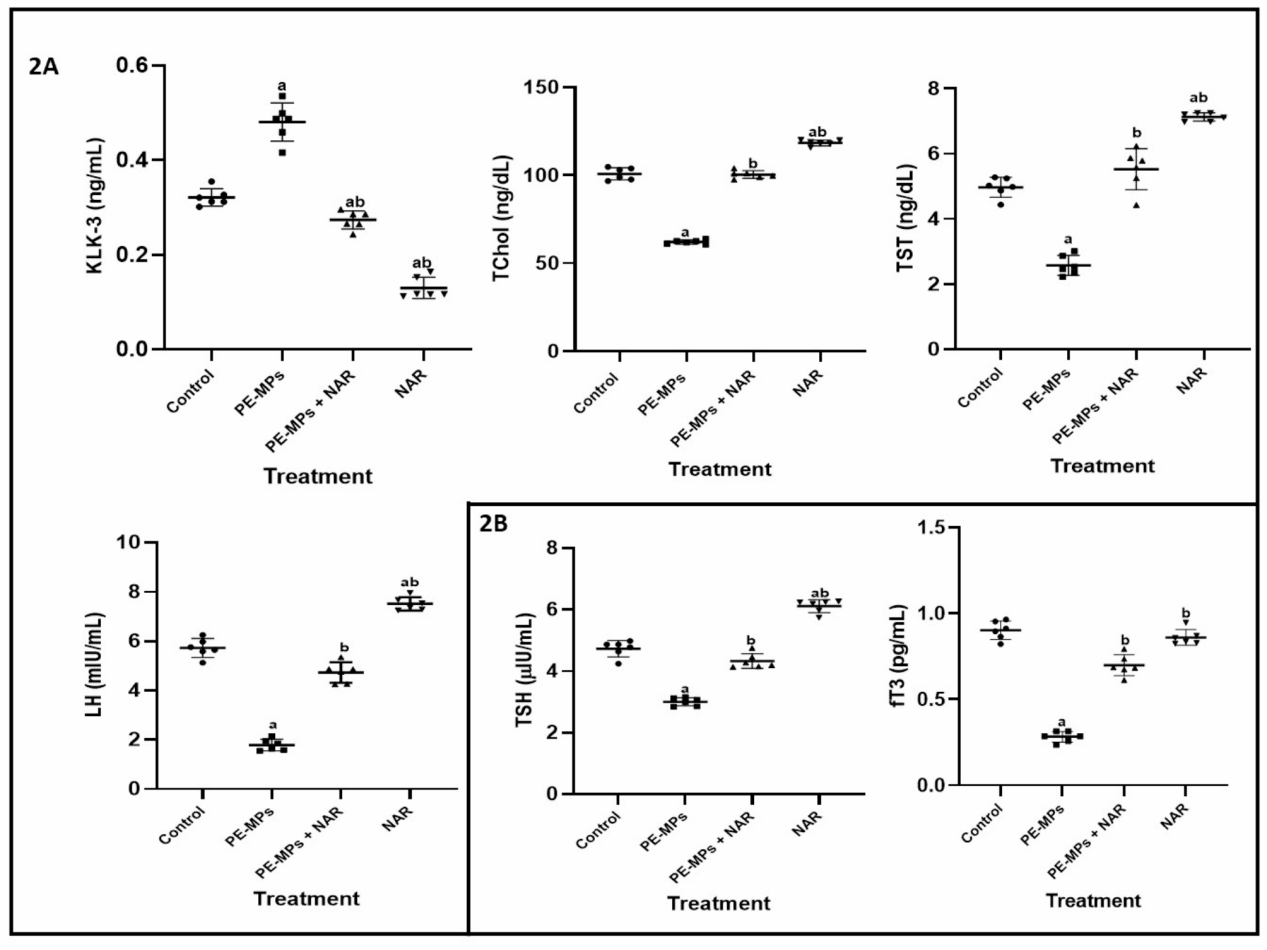
The ameliorative effect of Naringin on changes in the serum levels of KLK-3, TChol concentration, TST, LH, TSH and fT3 induced by PE-MPs in Wistar rats. Each bar represents the mean ± SD (*n* = 6). *PE-MPs *polyethylene microplastics, *NAR *naringin, *KLK-3 *prostate-specific antigen, *TChol *total cholesterol, *TST *testosterone, *LH *luteinizing hormone, *TSH *thyroid stimulating hormone, *fT3 *free-triiodothyronine, *SD *standard deviation, *n *replicates, a = significantly different (*P* < 0.05) from the control group. b = significantly different (*P* < 0.05) from the PE-MPs group.

The perturbating effect of PE-MPs and ameliorative potential of NAR on KLK-3, TChol, TST and LH is shown in Fig. 2A,B. Relative to control (Fig. 2A), PE-MPs significantly (*p* < 0.05) decreased KLK-3 level (59.50%) and TChol concentration (37.95%) in Wistar rats. Ameliorative potential of NAR on PE-MPs-induced alterations were seen in 52.23 and 99.38% increases in KLK-3 and TChol respectively. Exposure to PE-MPs elicited a significant decrease (*p* < 0.05) of 2-folds and 3-folds in TST and LH level respectively when compared with the control. However, co-administration of NAR subsequently ameliorated the decreases observed in the levels of TST and LH by 2 folds each. Figure 2B illustrates the alterations in levels of TSH and fT3 induced by exposure to PE-MPs and the modulatory potential of NAR on the alterations. TSH and fT3 level were significantly (*p* < 005) decreased by 36.23% and 68.88% respectively relative to control. These decreases were significantly (*p* < 0.05) reversed on co-administration with NAR by 30.23% and 71.87% (TSH and fT3) respectively.

**Fig. 3:**
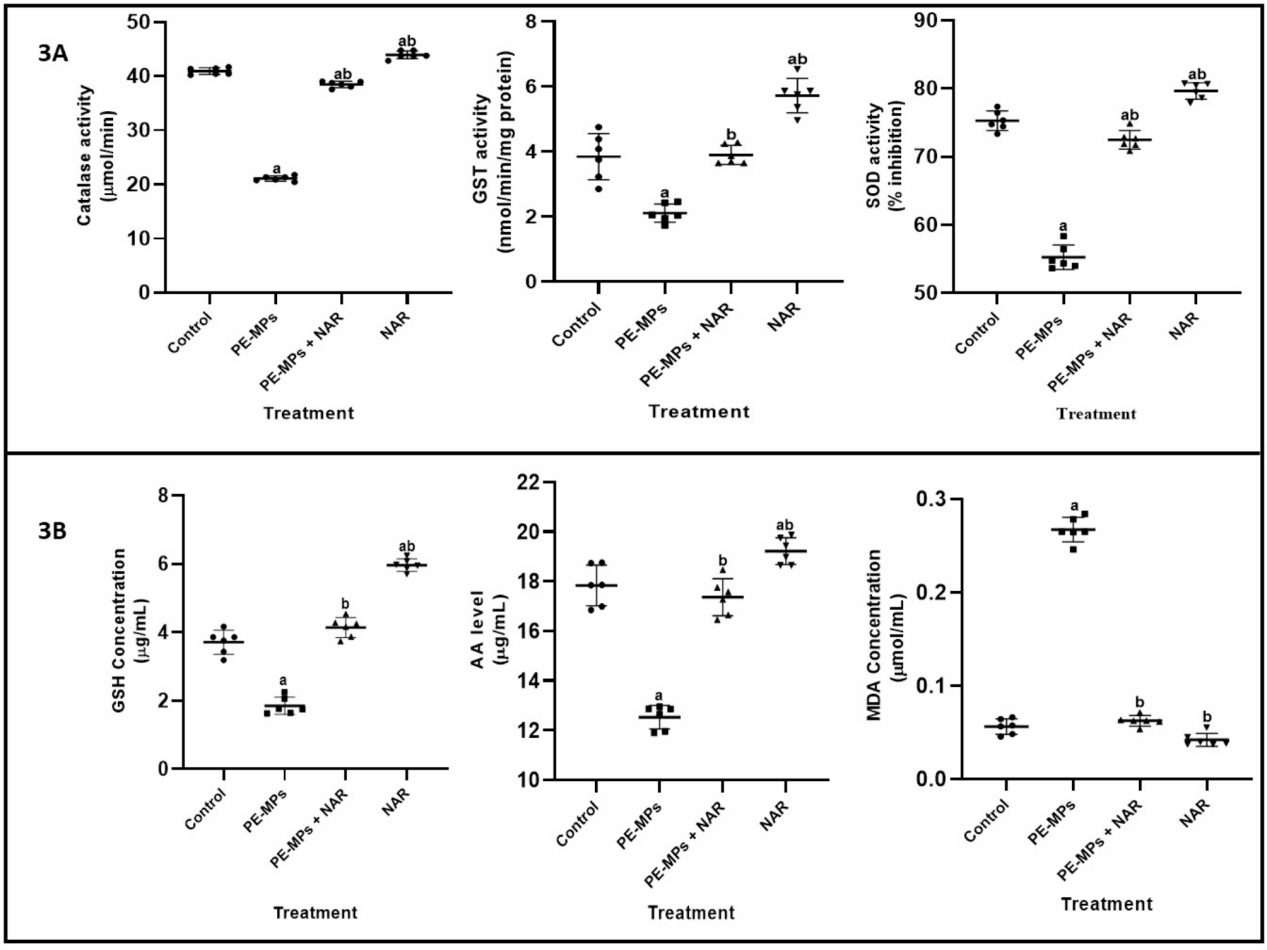
The ameliorative effect of Naringin on changes in the serum activities of CAT, GST SOD and levels/concentrations of GSH, MDA and AA induced by PE-MPs in Wistar rats. Each bar represents the mean ± SD (*n* = 6). *PE-MPs* polyethylene microplastics, *NAR *naringin, *CAT *catalase, *GST *glutathione-s-transferase, *SOD *superoxide dismutase, *GSH *glutathione, *MDA *malondialdehyde, *AA *ascorbic acid, *SD *standard deviation, *n *replicates, a = significantly different (*P* < 0.05) from the control group. b = significantly different (*P* < 0.05) from the PE-MPs group.

### Effects of PE-MP and naringin on serum antioxidants activities

The effect of PE-MPs and attenuating potential of NAR on the activities/levels of antioxidants (enzymic and non-enzymic) is shown in Fig. 3. Relative to control as seen in Fig. 3A, PE-MPs significantly (*p* < 0.05) decreased the activities of CAT, GST and SOD (48.48, 44.01 and 26.66 respectively) in Wistar rats. Ameliorative potential of NAR on PE-MPs-induced alterations in enzymatic enzymes activities were seen in 45.20, 54.23 and 23.77% increases (CAT, GST and SOD respectively) relative to control. Following exposure to PE-MPs, GSH concentration markedly (*P* < 0.05) decreased; however, this effect was reversed by 55.16% when Naringin (NAR) was co-administered. Moreover, Fig. 2B showed eight-fold increase (*P* < 0.05) in MDA concentration after exposure to PE-MPs, but substantially declined by seven-fold when concurrent NAR treatment was given. Lastly, PE-MPs exposure significantly decreased (*P* < 0.05) AA levels by 29.78%, whereas co-administration with NAR significantly increased (*P* < 0.05) AA levels by 27.88%.

## Discussion

The growing prevalence of plastic pollution in the environment has emerged as a major ecological and public health concern. Microplastics can enter the environment through multiple pathways, including the degradation of larger plastic debris, wastewater effluents, and industrial processes^29^. There is no universally “acceptable” level of microplastics in the environment, as their presence is typically seen as harmful due to potential negative impacts on ecosystems and human health. The concentration and effects of microplastics can differ based on environmental factors and specific conditions. Even small amounts can cause considerable ecological damage, such as ingestion by wildlife, physical injuries, and chemical pollution^30,31^. Thus, while a “safe” level of microplastics doesn’t exist, efforts are aimed at minimizing their presence and addressing their environmental impacts. Among these, polyethylene microplastics are especially common because polyethylene is widely used in numerous consumer products and packaging materials, such as plastic bags, bottles, and containers. Its durability and mass production contribute to the release of microplastics into the environment through degradation, making it one of the most prevalent forms of plastic pollution^32^.

Naringin, a flavonoid which very abundant in citrus fruits, has been reported for its anti-apoptotic properties, antioxidant and anti-inflammatory^8,33^. In this study, we investigated the ameliorative effects of naringin on disturbances in kallikrein-3 (KLK3) levels, steroid and thyroid hormones, and antioxidant status in a murine model exposed to polyethylene microplastics (PE-MPs). Our results demonstrate that exposure to PE-MPs significantly altered these physiological parameters, suggesting a potential risk posed by environmental microplastics. However, naringin mitigated many of these adverse effects, highlighting its protective role as co-administration of naringin in PE-MP exposed mice significantly ameliorated the alterations in KLK3 levels, hormone disturbances, and oxidative stress markers.

The primary role of KLK3, also known as prostate-specific antigen (PSA), is known to be in the male reproductive system. It is secreted by the epithelial cells of the prostate gland to liquefy semen in the seminal coagulum and allow sperm to travel freely^34^. It is a serine protease involved in various physiological processes, including inflammation and cancer progression. Elevated levels of KLK3 have been associated with health status of the prostate gland. Our study found a significant increase in KLK3 levels in the PE-MP exposed group compared to controls. This alteration suggests a possible inflammatory response or a disruption in normal proteolytic processes due to microplastic exposure. Elevated KLK3 levels may indicate a stress response in tissues, potentially leading to pathological conditions over prolonged exposure. Benign prostatic hyperplasia, prostate cancer, prostatitis are common conditions among others that can cause increase in KLK3 levels^35^. While a significant increase in KLK-3 levels can be concerning, it is not definitive for diagnosing prostate cancer, as various benign conditions can also cause KLK-3 elevation, However, Naringin treatment attenuated the surge in KLK3 levels, suggesting its role in modulating inflammatory responses and proteolytic activity. This protective effect could be due to naringin’s anti-inflammatory properties, which help maintain tissue homeostasis and prevent unnecessary activation of proteases. Naringin’s ability to modulate signaling pathways involved in inflammation, such as NF-κB and cytokine production, might play a crucial role in mitigating the inflammatory response and, consequently, the expression of KLK3^36^.

Cholesterol is a critical component of cellular membranes and a precursor for the synthesis of steroid hormones. It is also essential for maintaining cell membrane integrity and fluidity^37^. Disruptions in cholesterol homeostasis can lead to metabolic disorders, cardiovascular diseases, and other health issues. The notable reduction in TCHOL concentrations observed after PE-MPs exposure, as depicted in Fig. 2, compared to the control, may be attributed to the cholesterol-lowering effects of PE-MPs or changes in cholesterol production in the exposed animals. Additionally, PE-MPs exposure might have affected other biological molecules that depend on cholesterol as a precursor (e.g., bile acids), potentially redirecting available cholesterol for their synthesis. The normalization of cholesterol levels in naringin co-treated group points to the lipid-modulating effects of this flavonoid. It has been demonstrated that naringin alters lipid metabolism by up-regulating the activity of the enzymes involved in the synthesis and breakdown of cholesterol^38,39^. The underlying mechanisms may involve naringin’s capacity to modulate the activity of enzymes involved in cholesterol synthesis and catabolism, such as HMG-CoA reductase. Additionally, naringin’s antioxidant properties could protect against oxidative stress-induced lipid peroxidation, thus preserving the structural integrity of cholesterol and preventing its abnormal accumulation or depletion. The reduction in oxidative stress markers observed in the study further supports the hypothesis that naringin exerts a stabilizing effect on cholesterol levels by mitigating oxidative damage.

Exposure to PE-MPs resulted in significant perturbations in the levels of other key steroid hormones, including testosterone and luteinizing hormone. The disruption in steroidogenesis could be attributed to the endocrine-disrupting properties of microplastics and their additives, which can mimic or interfere with hormone receptors, synthesis and metabolism. The observed reduction in testosterone and LH levels suggests an anti-androgenic and anti-estrogenic effect, respectively, which may have far-reaching implications for reproductive health and development as size of microplastics being very small allows them to penetrate biological barriers, thereby potentially reaching the endocrine organs and disrupting their normal function. The Hypothalamic-Pituitary-Gonadal (HPG) Axis is critical in regulating the production and release of TST and LH^40^ and exposure to polyethylene microplastics and their associated chemicals can disrupt this finely tuned axis as EDCs has been reported to mimic or block the action of natural hormones, leading to altered GnRH, LH, and testosterone levels. Reduced LH levels can result in insufficient stimulation of the testes, thereby lowering testosterone production. Testosterone has roles beyond reproduction, including the regulation of muscle mass, fat distribution, and cardiovascular health^41^. Decreased levels of TST can lead to increased fat mass, reduced muscle mass, and a higher risk of metabolic disorders. Testosterone influences mood, energy levels, and cognitive functions. Reduced levels can contribute to fatigue, depression, and decreased quality of life^42^. Testosterone has been shown to have antioxidant properties. It helps in reducing oxidative stress by modulating the activity of antioxidant enzymes like superoxide dismutase (SOD) and catalase, which neutralize harmful reactive oxygen species (ROS). In males, testosterone’s antioxidant function is particularly important for maintaining reproductive health, as oxidative stress can negatively affect sperm function and testosterone production itself^43^. While LH itself may not have direct antioxidant properties, its regulation of sex hormones like testosterone and estrogen influences antioxidant activities as abnormal levels of LH can lead to hormonal imbalances that exacerbate oxidative damage. The studied reiterated the potential of Naringin supplementation to restore the levels of luteinizing hormone and testosterone closer to normal, indicating its role in protecting reproductive health and metabolic functions from the toxicity associated with PE-MPs due to its antioxidant and metabolic properties.

Thyroid hormones are crucial for metabolic regulation, growth, and development. In reaction to thyrotropin-releasing hormone (TRH) from the hypothalamus, the pituitary gland releases TSH^44,45^. It then stimulates the thyroid gland to produce the hormones T4 and T3. T4 is predominantly converted into T3, with a smaller proportion being converted into fT3, which is the active hormone that exerts metabolic effects. fT3 is the most active thyroid hormone, responsible for regulating various metabolic processes, including basal metabolic rate, lipid and carbohydrate metabolism, and thermoregulation^46^. Our findings indicated a significant decrease in thyroid stimulating hormone (TSH) and free-triiodothyronine (fT3) levels in the PE-MP exposed group. A decrease in TSH levels could indicate suppression of the HPT axis. This suppression can occur due to several factors, including negative feedback, direct inhibition, hypothyroidism and thyroid dysfunction suggesting that microplastics might disrupt thyroid function. Lower levels of fT3 can lead to symptoms of hypothyroidism, including fatigue, weight gain, cold intolerance, and slowed metabolism. These symptoms reflect the body’s reduced ability to utilize energy efficiently while it can lead to developmental delays and cognitive impairments. Also, Thyroid hormones has been shown to play a significant role in metabolism and energy regulation as thyroid hormones (regulated by TSH) influence mitochondrial activity and oxygen consumption, processes closely linked to oxidative stress. Imbalances in thyroid function (e.g., hypothyroidism or hyperthyroidism) can lead to increased oxidative stress, as mitochondrial dysfunction generates excessive ROS. fT3 enhances mitochondrial activity and oxygen utilization, processes that can generate ROS. However, it also boosts antioxidant defenses by promoting the expression of antioxidant enzymes. An imbalance in fT3 can lead to either excessive oxidative stress (hyperthyroidism) or insufficient metabolic activity and antioxidant defenses (hypothyroidism)^47–49^. Naringin effectively normalized the levels of fT3, and TSH. This suggests that naringin can counteract the endocrine-disrupting effects and oxidative distress potential of PE-MPs. The mechanisms may involve naringin’s ability to interact with hormone receptors, protect against receptor dysregulation, or modulate the synthesis, transport and metabolism of hormones through its antioxidant potentials among others. The restoration of hormone levels points to naringin’s potential in preserving endocrine function in the presence of environmental pollutants.

The induction of oxidative stress is a crucial mechanism by which microplastics exert their deleterious effects^12^. The oxidative stress markers, including MDA levels and the activities of antioxidant enzymes (GST, CAT and SOD), and non-enzymatic antioxidants (GSH and AA) were significantly altered in the PE-MP group. MDA, a byproduct of lipid peroxidation and also serves as a biomarker of oxidative stress^11,50^. Elevated MDA levels observed in PE-MPs-exposed mice indicate increased lipid peroxidation, suggesting significant oxidative damage to cell membranes. This increase in MDA reflects the pro-oxidant state induced by microplastics, which can compromise cellular integrity and function.

SOD and CAT are essential antioxidant enzymes that alleviate oxidative stress. SOD catalyses the conversion of superoxide radicals into O_2_ and H_2_O_2_, while CAT breaks down hydrogen peroxide into water and oxygen^51^. The decline in antioxidant enzyme activities of SOD, GST and CAT suggests a compromised antioxidant defense system, likely overwhelmed by the increased production of ROS due to microplastic exposure. GSH is a pivotal intracellular antioxidant that protects cells against oxidative stress by neutralizing ROS, thereby maintaining redox balance^52^. The significant reduction in GSH levels in the PE-MPs group indicates a depletion of this critical antioxidant, further highlighting the oxidative burden imposed by microplastic exposure. This oxidative stress can lead to cellular damage and inflammation, contributing to the overall toxic effects observed. The administration of naringin significantly reduced MDA levels and restored the activities of antioxidant enzymes. This indicates a decline in oxidative stress and an enhancement of the antioxidant defense system. Naringin’s ability to scavenge free radicals and upregulate antioxidant enzyme expression likely underlies these protective effects. By reducing oxidative stress, naringin helps protect cellular integrity and prevent inflammation and cellular damage.

The beneficial effects of naringin observed in this study can be attributed to its multifaceted actions. Naringin has been shown from this study to possess high antioxidant potential as it directly scavenges ROS thereby reducing the overall levels of oxidative stress within cells exposed to PE-MPs, boosts the activities of endogenous antioxidant enzymes like SOD and CAT, helping cells to counteract the oxidative distress induced by PE-MPs and in effect may help to maintain the balance between ROS generation and removal in the NAR-treated group. In the control group exposed to polyethylene microplastics, a pronounced oxidative stress response is typically observed. This is characterized by elevated levels of ROS, lipid peroxidation (as indicated by increased MDA), reduced antioxidant capacity (lower GSH levels), and possible mitochondrial damage. These markers reflect the significant cellular stress caused by PE microplastics. In contrast, the NAR-treated group likely shows a milder oxidative stress response. While microplastics exposure induce some level of stress, the antioxidant properties of Naringin help in reducing ROS levels, protecting cellular components from oxidative damage, and enhancing the activity of antioxidant enzymes. This would result in lower MDA levels and preserved mitochondrial function compared to the control group. Protective effect of Naringin is also seen in modulation of enzymatic activities and signaling pathways, and hormone receptor interactions. For example, naringin has been demonstrated to affect the nuclear factor erythroid 2–related factor 2 (Nrf2) signaling pathway, which plays a crucial role in cellular defense against oxidative stress^53^. By activating Nrf2, naringin could enhance the expression of various antioxidant enzymes, thereby bolstering the body’s defense. These actions collectively contribute to its protective effects against the toxic impacts of PE-MP exposure. The findings suggest that naringin could be a potential therapeutic agent in mitigating the health risks associated with microplastic exposure, particularly in mitigating endocrine and oxidative disruptions.

## Conclusion

In conclusion, this study underscores the potential risks associated with PE-MP exposure, highlighting significant disruptions in KLK-3 levels, steroid and thyroid hormones, and antioxidant status. It emphasizes the significant endocrine and oxidative disruptions caused by polyethylene microplastic exposure and highlights the potential of naringin as a mitigating agent. The results indicate that naringin’s antioxidant and anti-inflammatory properties are essential in safeguarding against the harmful effects of microplastics. Given the increasing prevalence of microplastics in the environment and their potential health risks, exploring natural compounds like naringin offers a promising avenue for mitigating these risks.

## Data Availability

The supporting data for this article are contained within the article itself, and the original study data of this study are available from the corresponding author upon reasonable request.
